# People’s perceptions and experience of managing life after recurrent pancreatitis: a qualitative study in eastern China

**DOI:** 10.1038/s41598-022-22287-w

**Published:** 2022-11-05

**Authors:** Lin Chen, Xingxing Zhou, Xiamin Tu, Hongmei Cheng, Zhaotao Duan, Guotao Lu, Yuan Yuan

**Affiliations:** 1 College of Nursing, Yangzhou University, Jiangsu, China; 2 College of Medicine, Yangzhou University, Jiangsu, China; 3 Department of Nursing, the affiliated hospital of Yangzhou University, Jiangsu, China

**Keywords:** Psychology, Gastroenterology, Health care

## Abstract

There is a high rate of recurrent hypertriglyceridemic acute pancreatitis (HTG-RAP) and risk of developing into chronic pancreatitis among recurrent hypertriglyceridemic acute pancreatitis. The key to avoiding recurrence is home-based self-management. However, self-management has proven to be difficult. Exploring experiences and perceptions of home-based self-management among patients with HTG-RAP could inform intervention development and policy making in primary care. To explore experiences and perceptions of home-based self-management among patients with HTG-RAP. This is primarily a qualitative study involving patients from eastern China. The study was designed using semi-structured interviews combined with open interviews among individuals and focus groups. Interviews with patients (n = 25) and relatives (n = 2) were conducted from October to December, 2021. Data were analyzed using the thematic analysis approach. Five themes were identified: (1) pity, (2) sense of uncertainty, (3) contradiction, (4) the way to cope, and (5) benefits. The themes constituted a continuous process where a final coping strategy was confirmed. Patients expressed sorrow, struggle, pity, adaptation, and benefits. The disease still bothered them without attack, both mentally and physically. These key points deserve considerable attention to improve the quality of life of patients and lifestyle modification. Patients with pancreatitis were more likely to manage the disease but under a tough process, and during the struggle, they experienced a continuous and contradictory period. Ultimately, the final condition was reached.

## Introduction

Acute pancreatitis (AP) is an inflammatory disease that can lead to severe consequences, including organ failure, systemic inflammatory response syndrome and local injury^1^. It is one of the most common gastrointestinal diseases that often present in most emergency departments. Mild AP, which is usually self-limiting and tends to recover in 3 to 5 days, has a mortality rate of less than 1%. However, the mortality rate of severe AP can reach 30–40%. According to a previous report, the prevalence of hospitalization for AP increased significantly in the United States from 2002 to 2012^2^. Clinical experience suggests that hypertriglyceridemic acute pancreatitis (HTG-AP) is associated with more severe outcomes than other etiologies^3^.

Hypertriglyceridemia can often lead to recurrent bouts of AP^4^. Among etiologies of pancreatitis, hypertriglyceridemia-induced etiologies often attack middle-aged and younger men^5^. Hypertriglyceridemia-induced AP increases the risk of local complications, recurrence, acute necrotizing pancreatitis, organ failure, and mortality and has a high prognostic score^6–8^. The clinical phenotype of recurrent acute pancreatitis (RAP) patients varies substantially, from asymptomatic conditions between episodes of AP to chronic symptoms between episodes^9^. Long-term outcomes of AP have gained more attention with the increasing survival rate after an AP attack^10^. Nevertheless, RAP patients have a high risk (10–40%) of developing chronic pancreatitis (CP) and its complications (e.g., declined quality of life) and the possibility of pancreatitis cancer^11^. Sometimes the quality of life may decline due to the occurrence of AP, which can be observed mainly in the following domains: physical function and social network, emotional role, and overall health^12^. The decline in the quality of life of patients can be caused by the adverse side effects of pancreatic dysfunctions, including continuous abdominal pain, malabsorption, diarrhea, unintended weight loss, new-onset pathoglycemia, and requirement for supplements of trypsin^10,12^. HTG-related risk factors include alcohol intake, diabetes, and hypothyroidism^13^, which are associated with an increased risk of obesity and metabolic syndrome^14,15^. The incidence of pancreatitis is higher in obese individuals and patients with obesity or metabolic syndrome tend to undergo a more severe course^16,17^. Because the aforementioned factors are lifestyle-related risk factors, proper self-management in daily life may help reduce the recurrence rate of HTG and improve the quality of life.

However, although patients pay significant attention to AP, a recurrence could be unintentionally triggered. Thus, paying special attention to the risk of RAP is a continuous but excruciating experience in this patient population.

AP is a severe inflammatory disease affecting patients both physically and mentally. Although the etiology and clinical treatment of AP have been well explored, studies assessing the rehabilitation process after pancreatitis attacks or the lived perception and experience from the patient’s perspective are scanty^18^.

A previous study investigated the emotional experience of AP patients in a hospital in China ^19^. Another study explored the experience of patients with CP in Ireland ^20^. A different study in Sweden evaluated the perception of patients recovering from AP using qualitative methods but only focused on the factors of recovery^18^. Generally, CP patients endured disruption and those in hospitals experienced hope, sorrow, and desire to overcome the disease. Patients recovering from AP underwent a tough journey both physically and emotionally and highlighted the significance of healthcare. In addition, few studies assessed the experience of AP patients in their routine life after recovery. Although management of pancreatitis becomes a daily routine, the perception of patients remains ambiguous. Herein, we sought to explore experiences and perceptions of home-based self-management among patients who experienced HTG-AP more than once.

## Methods

This study was registered at the Chinese Clinical Trial Registry (ChiCTR) on 27/06/2021 (Registration number: ChiCTR2100047871). All methods were performed in accordance with the relevant guidelines and regulations. The research was conducted by interviewing the participants and interviews were recorded after informed consent.

### Procedure

Face-to-face interviews with 25 patients who had experienced HTG-AP more than once and two relatives in the company of each patient were conducted. The study also included five focus groups (10 patients and 2 relatives) comprising two groups of the patient plus relatives and three groups of patients only were recruited. All interviews were held separately. The inclusion criteria were patients aged > 18 years and able to communicate clearly; those discharged from the affiliated hospital of Yangzhou University from May 2013 to July 2021. The exclusion criteria were patients who underwent surgery for pancreatitis once or had pancreatitis cancer or those whose last recruitment time was in the latest 3 months. Patients were invited to attend a gratis medical examination for a return visit. Appointment with patients was booked in advance on the phone. The items included 2-h postprandial blood glucose (2 h-PBG) and the interviews were carried out in a quiet meeting room during the break before the detection of 2 h-PBG. The semi-structured method was used to conduct the interview based on purposive sampling. At the beginning of the interviews, we revised the interviews guidelines according to the interactions with the first three patients. At the end of the session, open interviews were employed for data enrichment. One-to-one interviews were started with the question: ‘How is it going with your diet?’ to initiate the topic and motivate their interest. Focus group interviews began with the question: ‘Please talk about the influence pancreatitis exerts on you.’

### Design

All the study protocols were approved by the ethics committee of the Affiliated Hospital of Yangzhou University (2021-YKL06-09-008) and followed the consolidated criteria for reporting qualitative research (COREQ) checklist^21^.

All the participants signed informed consent to participate in the interviews. Interviews with patients were hosted by a researcher and audio-recorded after patients’ informed consent. The interviews lasted 30–80 min. The interviewer was a nursing graduate with clinical experience. During a daily work routine as a nurse, the interviewer’s interactions with patients provided a better understanding of patients’ requirements before the interviews. The items included in the guide are as follows: What changes has AP brought you both physically and mentally? How does AP influence your daily life, job, and social relationship? What do you think about the effects of lifestyle on health? How do you evaluate your management of AP and the condition compared with other patients with AP? What is your attitude towards the disease and life? What trouble or difficulties have you encountered during the management of AP? One-to-one interviews were exclusively between the moderator and the participant; an observer attended while the focus discussion was going on.

### Participants

A total of 18 semi-structured interviews and two open interviews were carried out from October to December, 2021. The duration after the first discharge ranged from 3 to 8 years. Demographic and baseline data, including age, occupation, marital status, education background, monthly income, offspring’s growth stage, frequency of recurrence, and history of intensive care unit (ICU) admission, were collected. Relative(s) who accompanied the patient to attend the appointment were also encouraged to join the interview, and considering the close family bond, intentional separation would lead to a conflict, which may upset family members. They might feel offended and the behavior released a signal of disharmony. That was embarrassing in China so we decided not to conduct separate interviews. In some cases, patients came to the meeting room at the same time, and they naturally formed a focus group. Sometimes, focus groups create a relaxing and safe atmosphere where touchy topics can be expressed freely. In focus groups, a general introduction to the interactive discussion was held to briefly explain the purpose of the research. All the interviews were audio-recorded under participants’ consent and transcribed verbatim within 24 h. No transcription of scripts was sent back to participants for correction or confirmation, and repeat interviews were not conducted. The moderator and other group members held a debriefing discussion every three weeks.

### Participant characteristics

A total of 25 patients with recurrent pancreatitis and two relatives participated. The demographic characteristics of all the participants are shown in Table 1. The two relatives were both patients’ wives. Considering that the number of relatives who accompanied patients was quite small, demographic information of relatives was not collected.

**Table 1:** Demographic Data ( N = 25).

	Mean (SD) or n (%)
Age	44.9 (8.2)
Gender (male)	22 (88)
**Living status**	
Alone	4 (10.8)^a^
In couple	20 (54.1)^a^
With parents	3 (8.1)^a^
With children	10 (27.0)^a^
**Education level**	
Primary school	1 (4)
Middle school	6 (24)
High school	3 (12)
Bachelor	14 (56)
Postgraduate	1 (4)
**Marital status**	
Single	2 (8)
Married	22 (88)
Divorced or widow(er)	1 (4)
**Stage of children**	
Elementary and secondary school or lower	7 (28)
College	5 (20)
Employment	11 (44)
Nullipara	2 (8)
**Monthly-income**	
< 1000	1 (4)
1000–2999	3 (12)
3000–4999	9 (36)
≥ 5000	12 (48)
Recurrent times	1.6 (1.3)
Last recurrent time (years ago)	2.7 (1.6)
ICU admission	3 (12)

^a^Multiple-choice question.

### Data analysis

The collected information was analyzed using inductive thematic analysis^22^, in which the majority of the themes and categories were determined by constant comparison or inductive reasoning. After transcription, the analyst made contact sheets in which general impressions and coding were recorded. Furthermore, memos were created for each interview and the analyst improved the memos at any time when new ideas emerged. The function of memos was to inform the analyst of what had come up, which helped maintain coherence. Codes that referred to the same phenomenon were clustered into categories that were aggregated into higher-level themes. Through this process, information saturation was achieved that no new data emerged and no further interviews were required. Codes were managed on paper without any software. A framework was developed where all themes and categories were classified.

### Ethics approval and consent to participate

The study has been approved by the Affiliated Hospital of Yangzhou University(2021-YKL06-09-008).

## Results

A total of 20 interviews with patients diagnosed with AP were conducted. An overview of the interviews is presented in Table 2. The duration after discharge ranged from 3 to 8 years and the frequency of recurrence ranged from 1 to 5 times. Of the 25 patients, three were admitted to ICU due to severe AP. Based on the lived experience and perspectives of people living with AP, five themes emerged and were considered significant aspects of self-management experience: (1) pity, (2) sense of uncertainty, (3) contradiction, (4) the way to cope, and (5) benefits. Saturation was established when no new themes or categories emerged.

**Table 2:** The groups of interviewees.

	Number	Relationship with the patient
One-to-one interview	15	
Focus group discussion among patients	3 (n = 2, n = 3 and n = 3)	
Focus group discussion with relatives	2 (n = 2 and n = 2)	Both wives

### Theme 1: pity-bemoan one’s fate

One of the essential themes generated from the analysis was the feeling of pity. Patients sympathized with themselves in the losses caused by pancreatitis. This theme was addressed mainly by middle-aged men who were burdened with the responsibility of supporting a whole family. Among women, the intensity of pity was not so strong. After a pancreatitis attack, patients took actions such as diet improvement, adequate rest, and abstinence from tobacco and alcohol to avoid recurrence. Consequently, their behavior was under rational management, not at their will. Moreover, the bond with previous friends was weaker than before, which led to a feeling of isolation.

“I cannot eat whatever I want. Because of my body, I can hardly drink (alcohol), which damages my business. I cannot support my family as well as before! Without alcohol, friends are not so close as before. Weaker and weaker.” (P16)

#### Having lost what was owned

In the past, the physiological function was intact and the body was strong enough to support their desire to fight. The gastrointestinal function had not been impaired; thus, participants enjoyed whatever they wanted, regardless of diet or health considerations. Nowadays, they are constrained to eat limited types of food, which resulted in the feeling of losing the right to enjoy delicious food and life. The influence is not only physical but also mental. Supposing they survived the disease’s attack during hospital stays and were free of complications or restrictions in daily life, the feeling of loss would be weaker. It is cruel to see something lost. Ruminating what has been lost, they tend to lament lost possession.

“Before, I could enjoy delicious food freely, did not need to consider what was healthy and what was unhealthy. After pancreatitis, my intestinal function was not as well as before. I love milk, but cannot assimilate it now.” (P3) “My body is weaker than before, and I don’t have the capacity to run ahead.” (P12)

#### Depression and frustration

Due to the requirements on lifestyle, especially diet, patients cannot attend dinners as often as before. Despite several attendances, they try to avoid taking alcohol or greasy food, contrary to people around them. As a result, friends reduce the number of invitations and patients gradually avoid having dinner with ‘normal people,’ which generated a sense of isolation. Disconnection with a social network is a source of pain. After a pancreatitis attack, the diminished body function reminded them of the realities of aging. Compared with a previous body condition, the somatic function is much worse. Body function and endurance were not as excellent as before, suppressing further development to achieve their life goals. They initially aimed high without the disease. In other words, their qualifications to strive and realize ambitious personal objects were deprived. It is the restrictions caused by pancreatitis that upset patients and therefore they regret what has been lost.

“In the past, I always drank with friends and talked about everything. But I hardly dine with them again because I cannot drink anymore. Sometimes, they do not restrict the topics after drinking, and it is not fit to be heard by sober men.” (P7)

#### Unable to support the family as expected

Some industries demand a culture of drinking, and alcohol is a tool to improve the relationship with cooperators; thus, the target projects get promoted. Patients with AP are limited to alcohol consumption, and in some way, their careers are impacted adversely. One distinct consequence is that they have few opportunities to get promoted, which may determine the ceiling of wages. Wealth is the root of making a living and supporting a family. Given these factors, AP patients can do nothing but regret it. The majority of participants were middle-aged men, the breadwinners of their families, to whom the responsibility for folks is a mission. The pain they are undergoing is inconceivable.

“Of course, the quality of life declined greatly. Income cannot rise because of my body. I cannot bear heavy loads. Thus, not much is used to support my family.” (P2)

### Theme 2: sense of uncertainty

#### Lack of control

The reason why participants adapt their lifestyle is that they want to gain control over their health. Even some of them would rather have the pancreas removed than bear the risk of a possible outbreak. Adequate knowledge helps ease the anxiety about the disease regardless of the aspects. If the desire to get AP controlled can be met, they are willing to sacrifice some valuable things. Patients expect a wonder drug to cure them of AP and prevent a recurrence.

Participants also expressed concerns about an uncertain future such as sudden deterioration. Patients wished that they paralyze their minds to prevent experiencing the severity. Temporary oblivion and evasion make them feel better, because they do need to consider the negative things.. Besides, some participants give in to the uncontrolled situation, giving up following a healthy diet, increasing alcohol intake, and ignoring harmful factors. Together, these factors reflect the significance of control.

“The disease seems to be out of control. Before my last recurrence, I did not eat greasy food, and gratuitously, it attacked me again. I am afraid someday, it will ‘blow up.’ Will pancreas removal help reduce the recurrence rate? I would rather pay for expensive treatments, only if it gets controlled.” (P1)

#### Worries about the outcome

Recurrent attacks of illness may evolve into CP, where patients have to bear continuous and endless suffering. Given the adverse effects of CP, the undetermined outcomes tend to greatly worry participants. In addition, the probability of developing cancer disturbs their mind, even though the odds are fairly low. Whereas the outcome is not under absolute control, the severe end is out of their means.

“Will it evolve into chronic (pancreatitis)? Will it become cancer? What will happen if I do nothing?” (P1, P7).

#### The curse of the disease

Participants highlighted the disturbance by AP in daily life. Everything they do must be taken into thoughtful consideration for the sake of recurrence prevention. Thus, they are invisibly surrounded by the disease and faced with a faceless enemy. The memories of hospital scenes were still fresh, and cannot be taken away. The thoughts might evolve into rumination during initial discharge. The recurrence of AP may occur at any time with no sign, bothering them anytime and anywhere. It was recognized that anything related to pancreatitis would alert them, which seemed an endless curse until the end of life. They were so sensitive to slight signals that any changes would convince them to suspect the possibility of an AP attack.

In terms of recurrence, patients expressed the desire to escape, the existence of a fierce threat, and the unwillingness to suffer the pain again. The influence brought such panic that what mostly motivates transition behavior was the desire to be free of AP. Although it is acute inflammation, the influence is lasting and frustrating like dark clouds surrounding them. Furthermore, the disease triggers are highly associated with daily lifestyle, permeating every aspect of one’s life. When a stomachache occurred, the patient was reduced to a nervous wreck, for fear of a probable AP attack. The pain was relieved with time and the tenseness was released. The more troubled by AP, the stronger the willingness to get rid of AP.

“Whenever my belly hurts, the worries about recurrence come to hit me. Then, I will eat nothing. It seems that the process is of waiting for recurrence. Several days later, as the pain released, my mental pressure got released too. […] It is bothering and besets me anytime and anywhere. It will stay with me lifelong. I desire to get rid of it!” (P8)

### Theme 3: contradiction

#### Faced with a dilemma when managing

Although great attention was paid to the daily diet, patients could not always obey the healthy rules and the outside world was not perfect or designed as patients expect. Sometimes they desired to do the proper things but reality did not allow it.

“People with pancreatitis understand everything that should be done but sometimes can do nothing. I know we ought to lead a healthy life, but I do not have spare time due to my job and cannot have a daily routine as expected. The majority of the time I am not home and spare little time with my family.” (P10)

#### Fight against appetite

Diet among patients with pancreatitis is strictly restrained, which demands them to control their diet. To conflict against appetite means going against the mechanism of the human body and a tough and grueling process. The difficulty in exercising was also mentioned frequently. The critical point lies in activating the action of exercising, brief but burdensome workouts. People chase after freedom and uninhibited condition, hence patients deprived of freedom in some way admire young men or healthy men without diet restrictions, which brings them sorrow.

“Faced with nice and yummy food, I cannot eat and have to starve. Excessively, when I go out for dinner with my children, I can eat just a little and watch my girls enjoy the meal. More importantly, the bill will be paid by me.” (P19)

#### Lack of knowledge

Patients do not know how to eat healthily and exercise effectively and require proper guidance on measures to prevent recurrence and keep fit. Patients realized the importance of management, and trying to take action, but lacked appropriate methods to achieve this. They noted that it would have been better if a personalized scheme contact with health management was offered. Thus, it can be concluded that knowledge is an aspect of great significance.

"Usually I cook at home, he (the patient) does not pay attention to diet. I don’t know what to eat and how to better prevent a recurrence. What can be eaten and what is not to be eaten?” (R1) “Is a little alcohol acceptable? If I exercise more, more drinking is ok, right?” (P10)

#### Objective conditions

At times, patients desired control over lifestyle but were bound by objective conditions such as parties being hard to turn down, the chef at home preferring unhealthier cooking, job scheduling, etc. According to patients, those hardships were hard and nearly impossible to overcome. Consequently, they had to sacrifice physical health in exchange for social network enhancement or career promotion.

“My father-in-law is a cook and he always cooks greasy and salty food and we cannot make him change the taste.” (P11) “Without alcohol, friends were further and some projects were hard to negotiate due to the ‘alcohol table culture’.” (P10)

#### Struggle

Participants stated that the fight with appetite was tough, they sought chances to satisfy a craving for good food. Rationally, they had better stay clear of what might worsen their health condition. While in such a stressed era, patients affirmed that everyone is burdened with heavy pressure, and therefore occasional indulgence helps in coping with stress and maintaining mental health and self-healing. Eating at their will was luxury and valuable, even though the adverse consequence was out of control. Thus, they were trapped in the torment caused by the conflicts between ration and sensibility. Sometimes, they had to choose between career and health. A current job could meet the demand to feed a family or emotional involvement made it tough to make up one’s mind. Besides, a minority denied the negative effects while some who depended on a company corroborated it. This reflected the phenomenon that they had self-esteem in that they refused to admit the terrible facts. Sometimes diet was not managed well but they chose to ignore the truth. Under such circumstances, participants blamed themselves for unfinished tasks and felt guilty for wrong behavior in the past and present.

“I am burdened with a mortgage. My son is young and he needs long support. In my company, I have to keep it running well in order to feed all the staff. The disease is contradictory to my life, but without current work, I will not manage to survive.” (P1) “He just denied the negative effects on his career, but in fact, he was influenced deeply. In the beginning, he was upset and shut himself at home. He is not willing to admit that.” (R2)

### Theme 4: the way to cope

#### Despair

Although patients were well aware of what to do, sometimes they could not do the proper things, leading to deep frustration. Some patients were bothered with metabolic syndrome, which made it difficult to improve their physical condition. In such cases, efforts might be in vain. In their opinion, lifestyle transition made no difference because though they did not do things beyond a certain extent or limit, AP would still recur. Therefore, there considered it useless, which led to despair. What made them even more resentful were those people who lived carelessly received fewer attacks. All the above-pushed patients into despair.

“Trying to manage will not be of use. It will come without signals and regardless of patients’ behavior.” (P22)

#### Adaptation

Since AP was a traumatic event among patients, the sequence of important items in life was changed. For instance, before AP, patients put too much emphasis on wealth, but after experiencing AP, they realized the value of life and health. The innermost desire and self-belief, as well as perception of life, were refreshed. In terms of diet, the feeling of slight hunger but not satiety was underscored, along with greasy diet aversion. The loathing was both physical and mental, and patients developed the ability to refrain from desire. Attitudes towards life changed delicately, patients did not render service to work regardless of cost. They treasured other things. Finally, a compromise between life and disease was reached that despite the unpredictable end of life, patients ought to make full use of every day before the end and face death peacefully.

“Now I feel sick of the oleaginous if only one glance at it. To live longer, we adjust eating habits, transform dislikes into likes, and chase the sense of hunger. Once, I was quick-tempered and did my best in work and so emulous, but now, I have changed.” (P23) “At such an age, we will come to the end of life and face death someday. The end is unknown but close, I can do nothing but prolong the end by proper lifestyle.” (P17)

### Theme 5: benefits

Everything has two sides and terrible experiences can stimulate positive thinking. Invisible diseases, such as fatty liver, have been detected following hospital stays, contributing to health promotion. As a result of the behavioral transition, some patients successfully lost weight, living up to the unreachable aims in the past. Additionally, the pain patients suffered alerted relatives of the consequences of an unhealthy lifestyle; thus, dependents modified their behavior. Meanwhile, patients gained more attention and care. In terms of friends, patients recognized that AP was a chance to filter friends, real friends left and this provided an opportunity to rethink life.

“Surely, my family modified lifestyle after seeing my miserable experience, which is a benefit.” (P17) “During the hospital stay, fatty liver was detected, which could not be detected at ordinary times.” (P4) “With diet management after discharge, I successfully lost weight. You know, it was fairly tough. Everything has two sides and is not an exception, it has both advantages and disadvantages. My attitudes to life and health changed, treasuring life more, and recognized what is really valuable, paying less attention to unmeaningful things.” (P12)

## Discussion and implications

**Figure 1:**
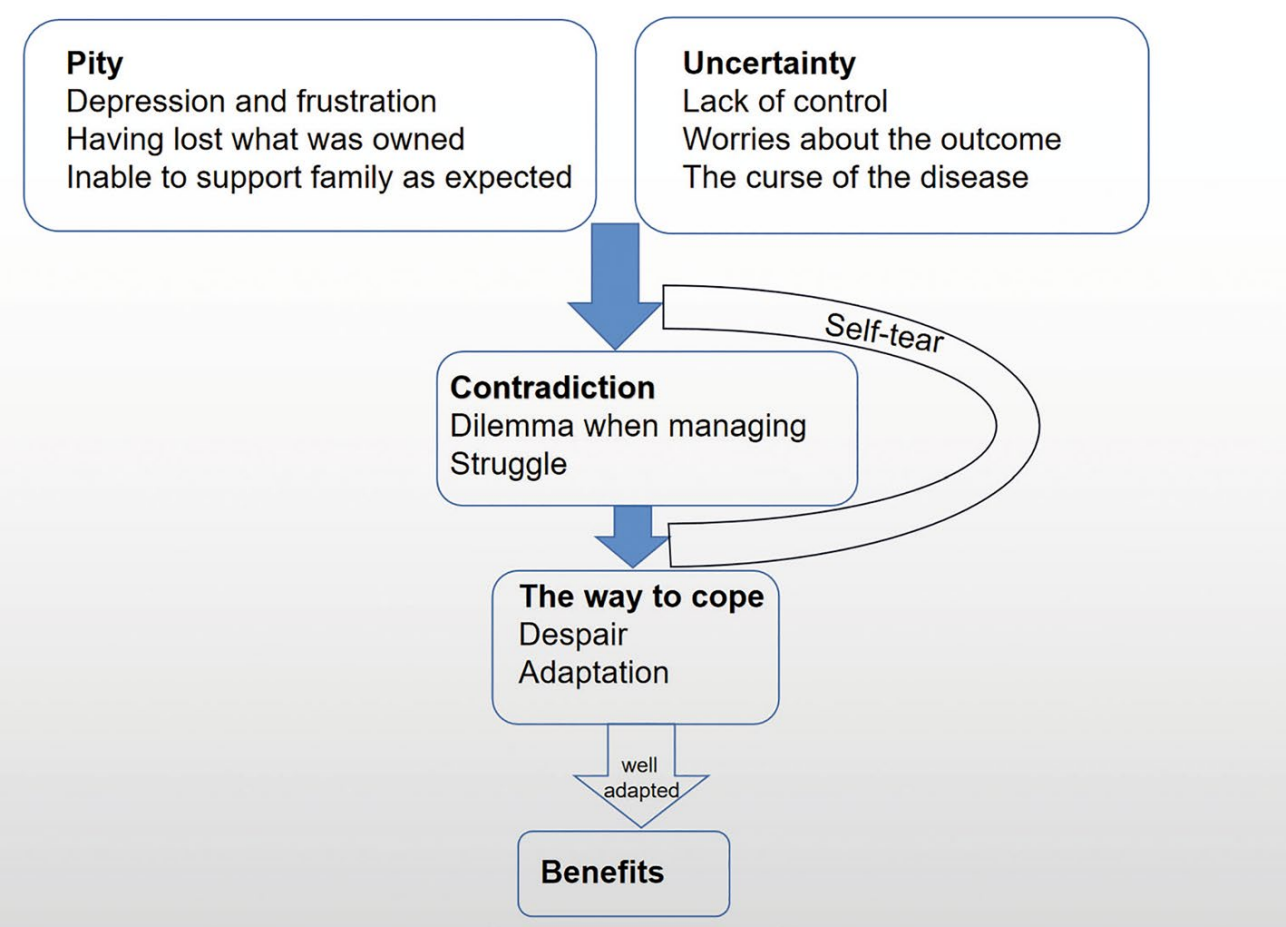
The process of people’s adaptation to self-management. Patients tended to experience pity and uncertainty at the beginning and as the process went on, they gradually felt contradiction. The pain promoted self-tear. During the process of self-tear, they would adapt to life in two different ways: failed to manage life well and felt despair or positively adapt to life well and get benefits. The despair might be temporary and they would adapt well finally and get benefits after proper attempt.

This study revealed the following five themes concerning patients’ experience in: (1) pity, (2) uncertainty, (3) contraction, (4) response, and (5) benefits. The five themes seem to form a continuous causal relationship. The generation of AP stimulated a series of responses and adaptions, and changed the pattern of coping. During the process, participants experienced sudden hit and then concentrated on improving the recovery of AP and acceptance of the reality that disease management is lifelong process. Such patients went through a difficult phase before obtaining a final coping mechanism. A framework of the process is shown in Fig. 1.

As is shown in Fig. 1, at the beginning of self-management, patients tended to experience negative feelings such as pity, sense of uncertainty, among others. Over time, some people achieved better results, realized the significance of effective self-management and gradually developed positive emotions. For others, the results were not satisfactory and they gave up self-management process. The duration of struggling is called ‘self-tear’ in this study. Once self-tear was completed, the final coping strategy was determined. Patients might adapt to a new life well and get benefits or cannot manage the life well and trapped into despair.

Almost all patients realized the significance of self-management however they did adhere to the management programs partly due to dissatisfaction with disease progress. According to Brooks et al.^23^, hope is influenced by the individual’s perceived threats on the horizon. Patients struggled to maintain a balance between despair and hope. A study by Mezzina^24^ reported that disease control is an important element during the recovery process and it can be gained if patients have the capacity to deal with the signs and symptoms of the disease. People chase for the sense of control and it can encourage people’s behavior. Without inspiration by control, they found it a struggle to insist on burdensome self—control, exhausted. Eventually, they abandoned. In such way, the coping style is negative. Seligman and Maier noted that people trapped in uncontrollable conditions, their future exploration and learning is impaired, which results in learned helplessness, a state where people cannot manage to take control due to lack of exploration^25,26^. This highlights the significance of control. As a label attached by social network, stigma is perceived. People’s negative mental encounter along with repeated label experiences seem to strengthen the attached label and incorporate it into the individual's identity^24^. Sometimes, patients are labeled the note of hedonism and lacking self-control and want to remove it. This process is referred to as self-tearing. The reasons why the patients gave up were not absolutely due to despair caused by learned helplessness. Everyone behaves motivated. Besides despair, they think of extravagant life as a sign of success, ignoring its harm to body. Adopting an unhealthy but “successful” diet satisfied their needs for respect. For instance, they paid attention to others’ opinion about them, therefore became complacent with their living situation, because extravagant diet meant high status. What accounted for the phenomenon was that they might not hold their confirmed values and weak self-conscious, following the society’s mainstream appraisal system. Sometimes the irrational behavior reflects unsatisfied needs. According to the self-determination theory^27^, three specific psychological nutrients are required to thrive which include relatedness, competence, and feelings of autonomy. Competence refers to perceived capacity to deal with the environment effectively. Autonomy is the ability to make decisions in one’s preferred perspective. Self-determination theory states that, as time passes by, unmet needs will cause a particular type of compensatory behavior, namely, instead of unfulfilling material or external needs, searching for satisfaction rather unfulfilling ^28,29^. Therefore, we have to find the key root if meaning to influence the patients and help with behavior modification. Understanding unmet needs will help clarify the underlying causes of intricate facts. As mentioned above, interventions should be directed at individual characteristics and aim to improve autonomy.

Besides, a group consisting of similar patients who share semblable experiences will work. Thus, the points above should be addressed to the change patient’s daily routine.

Lifestyle mirrors the way people think and their values in some cases. When it comes to things closely associated to family and job, all is uneasy and knotty. The patients were not able to balance between lifestyle and career routine. Choosing well-paying jobs meant that the individuals will have higher ability to provide for their families. However, this will mean that they will have a higher the risk of disease attack. It was always a single choice and hardly mutual victory.

We speculate that patients were motivated to modify their lifestyle but constrained by reality conditions around them. Besides, professional knowledge is required to help them manage their daily lives. To achieve effective adaptation, patients require targeted assistance, from knowledge guidance to motivation stimulation. Overall, general guidance combined with personalized implications will be of use and emotional fluctuate deserves attention, which demands long-term and reflective spiral of progress. Moreover, individual guidance should to be taken into consideration in primary care and prevention.

### Strengths and limitations

One obvious advantage of our research is that the recruited participants underwent free medical examination by a team of medical postgraduates specialized in acute pancreatitis. Therefore, the patients tended to show great interest in our interview. Moreover, they did not scruple when expressing themselves and largely relied on research team, therefore the information obtained was credible. However, the majority of pancreatitis patients were middle-aged men, hence the women’s perceptions were not deeply investigated. Due to time constraints, we did not return the transcriptions to participants. Another possible limitation is the distortion of meaning following language translation into English.

## Conclusion

Lifestyle mirrors the way people think and their values in some cases. When it comes to things closely associated to family and job, all is uneasy and knotty. The patients showed indecisiveness in balancing lifestyle and career routines. Choosing well-paying jobs meant that the individuals will have higher ability to provide for their families. However, this will mean that they will have a higher the risk of disease attack. It was always a single choice and hardly mutual victory.

Above all, it can be summed that patients were motivated to have lifestyle modified but constrained by reality conditions around them. Besides, professional knowledge is also required necessarily to better manage their daily life. To achieve the purpose of proper and effective adaptation, patients need targeted assistance, from knowledge guide to motivation stimulation. On the whole, general guide combined with personalized implications will be of use and emotional fluctuate deserves attention, which demands long-term and reflective spiral of progress. What’s more, the individual guide ought to be taken into consideration about primary care and prevention.

Based on the attitudes and perspectives of people with pancreatitis, we conclude that to better improve a person with pancreatitis, it is significant to overcome the ineradicable barriers, during which process confidence gets curtailed frequently and timely encouragement to rebuild confidence is necessary. The four themes seem to form a continuous and causal process, and each phase is highly related to the previous one. The results mentioned above provide a possible reference for developing interventions to assist pancreatitis patients with lifestyle modification.

## Data Availability

The datasets generated and analysed during the current study are not publicly available due to participants’ privacy but are available from the corresponding author on reasonable request.

## Abbreviations

AP: Acute pancreatitis
CP: Chronic pancreatitis
HTG-AP: Hypertriglyceridemic acute pancreatitis
HTG-RAP: Hypertriglyceridemic recurrent acute pancreatitis
RAP: Recurrent acute pancreatitis
